# Green Tea Polyphenol Nanoparticles Reduce Anxiety Caused by Tobacco Smoking Withdrawal in Rats by Suppressing Neuroinflammation †

**DOI:** 10.3390/toxics12080598

**Published:** 2024-08-18

**Authors:** Alaa M. Hammad, Lujain F. Alzaghari, Malek Alfaraj, Vanessa Lux, Suhair Sunoqrot

**Affiliations:** 1 Department of Pharmacy, Faculty of Pharmacy, Al-Zaytoonah University of Jordan, Amman 11733, Jordan; 2 Department of Genetic Psychology, Ruhr University Bochum, 44801 Bochum, Germany; vanessa.lux@rub.de

**Keywords:** anxiety-like behavior, BDNF, green tea, nanoparticles, neuroinflammation, neuroplasticity, NF-ĸB

## Abstract

Repeated exposure to tobacco smoke causes neuroinflammation and neuroplasticity, which correlates with smoking withdrawal-induced anxiety. The purpose of this study was to investigate the anticipated involvement of antioxidant-rich nanoparticles (NPs) prepared by oxidation-triggered polymerization of green tea catechins in impacting these effects in a rat model of tobacco smoke exposure. Exposure to tobacco smoke was carried out for 2 h a day, 5 days a week, for a total of 36 days. Weekly behavioral tests were conducted prior to recommencing the exposure. Following a 20-day exposure period, rats were administered either distilled water or green tea (GT) NPs (20 mg/kg, orally) for an additional 16 days. Our findings revealed that tobacco smoke exposure induced anxiety-like behavior indicative of withdrawal, and this effect was alleviated by GT NPs. Tobacco smoke exposure caused a marked increase in the relative mRNA and protein expression of nuclear factor-kappa B (NF-κB) and reduced the relative mRNA and protein expression of brain-derived neurotrophic factor (BDNF) in the hippocampus (HIP) and hypothalamus (HYP) brain subregions. The intervention of GT NPs effectively inhibited these effects. Our findings demonstrate the potent protective role of GT NPs in reducing withdrawal-induced anxiety-like behavior, neuroinflammation, and neuroplasticity triggered by tobacco smoke exposure.

## 1. Introduction

Tobacco consumption stands as a fundamental factor contributing to preventable illnesses and premature mortality on a global scale [1]. Tobacco products are among the most widely used psychoactive substances internationally [2]. The anxiety resulting from quitting smoking, as well as anxiety triggered by smoking, may influence smoking behavior, and research on the association between tobacco usage and anxiety conditions has yielded conflicting findings [for review, see [3]]. Past research indicates that anxiety serves as a fundamental and quantifiable symptom of nicotine withdrawal [4,5,6]. Previous clinical research has confirmed the contribution of mesolimbic dopamine transmission to anxiety-related processes in the nucleus accumbens [7,8]. Additionally, research on chronic stress has shown that increasing levels of oxidative stress indicators are connected with an increase in anxiety-like activity in various mesolimbic circuitry regions [9]. Furthermore, nicotine withdrawal results in proinflammatory effects, anxiogenic behavior, and morphological changes in microglia [10]. Regarding potential molecular correlates of these effects, nuclear transcription factor kappa B (NF-κB) and brain-derived neurotrophic factor (BDNF) have been linked to these processes. Specifically, NF-κB is activated by exogenous oxidants from tobacco smoke, promoting the formation of reactive oxygen species (ROS) and proinflammatory mediators [11,12]. Furthermore, mechanisms governing neurological development, neuroplasticity, and damage recovery interact with neuroinflammatory mediators [13]. In fact, the inflammatory state induced by lipopolysaccharide (LPS) injection in mice decreases the amount of the BDNF in the prefrontal cortex (PFC) and hippocampus (HIP), while it induces level of BDNF in the nucleus accumbens (NAc) [14]. This is supported by the finding that adult rats given intra-hippocampal BDNF experience a decrease in anxiety measured by the elevated plus maze test [15].

Plant polyphenols are secondary metabolites of plants that have demonstrated an extensive array of biological activities [16]. Green tea, which is one of the types of tea prepared from *Camellia sinensis*, is a widely consumed beverage that has a variety of pharmacological benefits, including antimutagenic, antiproliferative, and anticarcinogenic effects. Importantly, tea is the most widely consumed beverage in the world, second only to water [17]. Polyphenols in green tea, namely, (-)-epigallocatechin gallate (EGCG), (-)-epicatechin gallate (ECG), (-)-epigallocatechin (EGC), and (-)-epicatechin (EC), are assumed to be responsible for these properties [18]. Importantly, green tea has been shown to be neuroprotective in models of degenerative diseases [19,20,21]. According to a study conducted on rats, polyphenols unique to green tea may offer the ability to shield against neuronal cell loss in Parkinson’s disease [22]. Additionally, green tea demonstrates anti-inflammatory qualities. In a previous study, catechins were able to reduce the inflammation brought on by the anticancer agent doxorubicin. In addition to their numerous health benefits, green tea polyphenols have recently been explored as nanoparticle (NP) precursors [23,24,25]. This is attributed to the ability of these chemically versatile compounds to undergo oxidation-triggered self-polymerization under various conditions. Similar observations were reported for coffee bean [26] and grapeseed extracts [27], as well as individual polyphenolic compounds such as quercetin [28,29,30,31].

Anxiety caused by withdrawal is frequently examined in rodents using the elevated plus maze (EPM), open field (OF), and light and dark (LD) tests [32,33,34]. We have recently shown that NPs formed by spontaneous oxidation of dark-roasted coffee bean extracts could mitigate the behavioral as well as the molecular effects of withdrawal from cigarette smoking in mesocorticolimbic brain subregions (nucleus accumbens and prefrontal cortex) of female rats upon administration for 21 days [35]. This led us to investigate for the first time the effect of NPs derived from green tea antioxidants (GT NPs), which contain more antioxidants than the nanoassemblies from dark-roasted coffee bean extracts, in a similar animal model. Moreover, we investigated the effect of GT NPs on neuroinflammatory markers such as NF-κB and BDNF in the hippocampus (HIP) and hypothalamus (HYP) brain subregions. Through a combination of behavioral and molecular investigations, we present novel evidence regarding the role of antioxidant-rich GT NPs as a therapeutic intervention for neuroinflammation and anxiety-like behavior induced by tobacco smoke exposure during smoking withdrawal periods.

## 2. Experimental Section

### 2.1. Materials

A local distributor (Amman, Jordan) provided LD blue cruise cigarettes (Liggett Ducat, 0.6 mg nicotine, 0.8 mg tar, and 0.01 mg carbon monoxide). Green tea polyphenol extract containing at least 60% total catechins (Polyphenon 60) and copper sulfate (CuSO_4_) were acquired from Sigma-Aldrich (St. Louis, MO, USA). Regenerated cellulose dialysis membranes with 12–14 kDa molecular weight cut-off (MWCO) were acquired from Repligen (Billerica, MA, USA).

### 2.2. Preparation and Characterization of Green Tea Nanoparticles (GT NPs)

Green tea (GT) NPs were produced by oxidative coupling assembly of green tea polyphenols using the method described by Chen et al. with a few modifications [25]. One hundred milliliters of distilled water were heated to 50 °C in a 250 mL round-bottomed flask, to which 1 g of Polyphenon 60 and 100 mg of CuSO_4_ were added and mixed until completely dissolved. The solution was vigorously stirred protected from light for 3 h at 50 °C. The flask was then left at room temperature for 1 week. GT NPs were purified by membrane dialysis (12–14 kDa MWCO) against deionized water, and the medium was changed twice a day for 1 week. The NPs were then kept at 4 °C for further analysis.

### 2.3. Characterization of GT NPs

Particle size, polydispersity, and zeta potential of freshly prepared GT NPs were measured by dynamic light scattering (DLS) using a Nicomp Nano Z3000 instrument (Entegris, Billerica, MA, USA). Samples were prepared and evaluated as previously reported [36] and results are expressed as the mean ± standard deviation (SD) of at least three different NP batches. The morphology of the NPs was examined by scanning electron microscopy (SEM) utilizing a Zeiss Sigma VP field-emission scanning microscope (Carl Zeiss AG, Oberkochen, Germany). One drop of NPs was pipetted on a mica substrate and air-dried, followed by sputter-coating with platinum. Images were acquired at an accelerating voltage of 6 kV. GT NPs were also characterized by FT-IR and UV-vis spectroscopy as previously described [26].

### 2.4. Antioxidant Activity of GT NPs

Antioxidant activity of the NPs was evaluated by a 2,2-diphenyl-1-picrylhydrazyl (DPPH) assay as previously reported [36]. Briefly, the DPPH reagent (Sigma-Aldrich) was prepared as a 0.1 M solution in ethanol. Polyphenon 60 and GT NPs were each diluted in distilled water to obtain 1000, 500, 250, 125, 62.5, and 31.25 µg/mL. Then, 4 mL of the DPPH mixture was mixed with 250 µL of each sample and incubated in the dark for 30 min. Samples’ absorbance was recorded at 517 nm and compared against the blank (4 mL DPPH and 250 µL distilled water). Antioxidant activity was then calculated according to the following equation:

$$Antioxidant\;activity\;\%=\frac{\left(A_{blank}-A_{sample}\right)}{A_{blank}}\times 100\%$$

where *A_blank_* and *A_sample_* are the absorbance of the blank and the sample at 517 nm, respectively. Results are stated as the means ± SD for three different NP batches.

## 3. Animals

For this study, twenty-four male Sprague-Dawley rats (200–225 g) were used, which were inbred at Al-Zaytoonah University of Jordan (ZUJ). The rats were kept in a climate-controlled vivarium on a reversed 12 h light–dark cycle. In the home cage, each cage contained four animals, and there was unrestricted availability of food and water. The living conditions and care practices for the animals were in agreement with the Helsinki guidelines for animal research [37], and the Institutional Animal Care and Use Committee at ZUJ gave its approval to the study’s protocol (approval 3/1/2020–2021).

### Tobacco Smoke Exposure Animal Model

**Figure 1:**
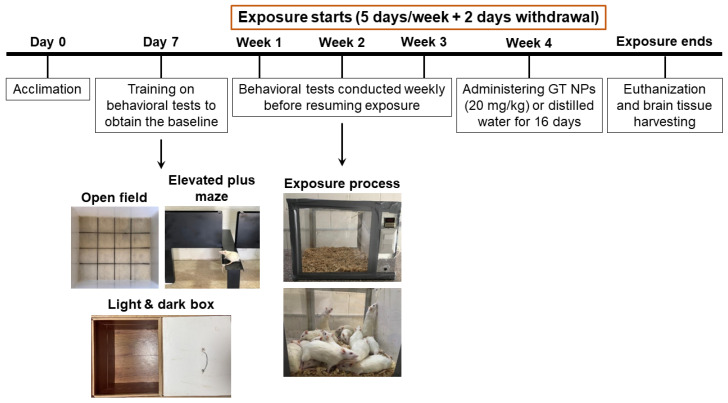
Timeline of the experiment involving tobacco smoke exposure, GT NP intervention, and behavioral testing.

As illustrated in Figure 1, the study timeline encompassed behavioral assessments, administration of GT NP intervention, and exposure to tobacco smoke. The study consisted of four groups that were assigned randomly, taking into account comparable baseline behavior: (1) the control group, which remained in a standard air environment for 36 days and received distilled water orally during the final 16 days; (2) the CIG group, which was exposed to tobacco smoke for 36 days and received distilled water orally during the final 16 days; (3) the GT NP (GT) group, which remained in a standard air environment and received 20 mg/kg GT NPs p.o. for the final 16 days; and (4) the CIG+GT group, which was exposed to tobacco smoke for 36 days and received GT NPs (20 mg/kg, p.o.) 45 min before exposure during the final 16 days. The GT NP dose was chosen according to our previous study using coffee bean aqueous extracts [35].

During each exposure session, rats were placed in a specially designed acrylic box (40 × 40 × 40 cm; Figure 1) for 2 h. Rats from the CIG and CIG+GT groups were exposed to tobacco smoke for 2 h, which was drawn from burning cigarettes through a pump and introduced into the exposure box. The pump operated on a repeated cycle involving a 3-second puff with a 30-second interval between the puffs for the duration of the exposure, which was set according to a previous study [38]. At the beginning of the exposure, two cigarettes were used to saturate the exposure chamber after the rats were placed inside, then the cigarettes were configured to burn for 3–5 min each. Twelve cigarettes were used within the 2 h exposure session. Carbon monoxide (CO) levels in the exposure chamber were monitored using an electrochemical sensor (Monoxor Ⅱ, Bacharach Inc., New Kensington, PA, USA). The CO meter readings in this experiment ranged from 600 to 800 ppm to make sure that the rats had taken an enough amount of nicotine without suffocating, which was verified previously in our lab by measuring cotinine (the predominant nicotine metabolite) in rats’ plasma [39].

## 4. Behavioral Tests

At the beginning of the study, behavioral tests were carried out and rats were assigned to the different groups to ensure similar baseline behavior in each group. Then, 24 h following the conclusion of each week of tobacco smoke exposure (2 h a day, 5 days a week) during the 2-day withdrawal period, behavioral tests (open field, OF; light and dark box, LDB; and elevated plus maze, EPM) were performed for the control, CIG and CIG+GT groups. As for the GT group, behavioral tests were conducted before and after 16 days of GT NP intervention. An observer who was not knowledgeable of the aim of the experiment recorded the results of the behavioral tests. Animals were permitted to habituate to the test apparatuses for 10 min before starting each test session, and the apparatuses were thoroughly cleaned with water between sessions.

### 4.1. Open Field (OF) Test

A white plastic enclosure functioned as an open field (OF) apparatus following a previously described procedure [33]. Measurements were taken for the total distance covered and the duration spent in the central zone during each 20 min session.

### 4.2. Light and Dark Box (LDB) Test

The light and dark box (LDB) apparatus consisted of two linked compartments: one was illuminated by a table lamp (considered the aversion region), and the other was kept in the dark (serving as the safe area), with a 7.5 cm × 5 cm opening connecting them [34]. A camera mounted on a tripod was placed above the LDB. Rats were initially placed in the illuminated compartment, away from the opening, to explore the perimeter until the entrance to the dark compartment was located. The duration spent in the illuminated compartment was then recorded.

### 4.3. Elevated Plus Maze (EPM) Test

The elevated plus maze (EPM) was composed of two open arms (without walls; 50 cm × 10 cm) and two closed arms (with walls; 50 cm × 10 cm × 30 cm), arranged in a cross shape with a central zone located approximately 50 cm above the ground, as previously detailed [32]. Rats were positioned in the center of the EPM, facing an open arm, and permitted a 5 min exploration period. Throughout this duration, both the number of crossings across the central zone and the time spent in the open arms were observed and recorded.

### 4.4. Brain Tissue Harvesting

Rats were euthanized using diethyl ether and cervical dislocation 24 h after completing the final behavioral tests. Subsequently, brains were promptly extracted, flash-frozen in liquid nitrogen, and kept at –80 °C. The HIP and HYP regions were later defined employing a cryostat (JUNG CM 1500, Leica, Wetzlar, Germany, Serial #043626787) maintained at −20 °C. The Rat Brain Atlas served as a reference for precise identification of distinct brain areas [40]. The dissected brain tissues were stored at –80 °C for later examination.

### 4.5. Real-Time Quantitative PCR (RT-PCR)

RNA extraction from the HIP and HYP regions was executed employing a Zymo Research kit (cat# R2052). A PrimeScript RT Master Mix kit from Takara Bio (Japan) was employed for the reverse transcription of RNA samples from both the HIP and HYP into cDNA. Refer to Table 1 for the primer sequences employed in the RT-PCR analyses. RT-PCR was conducted using a Prime-Pro iCycler instrument (Cole-Parmer). To evaluate the relative mRNA gene expression in comparison with the internal control for each gene, the mRNA expression recorded in each sample was standardized to β-actin mRNA, and the relative mRNA gene expression versus the internal control was quantified by determining 2^−ΔΔCT^ for each sample [41].

### 4.6. Western Blot

The HIP and HYP brain regions were homogenized with a standard lysis buffer. Similar amounts of isolated proteins were mixed with a 5X Laemmli loading dye. Protein separation was achieved through sodium dodecyl sulfate–polyacrylamide gel electrophoresis (SDS-PAGE; 10%), followed by electrophoretic transfer onto a polyvinylidene difluoride membrane (PVDF, cat# sc-3723) using a Trans-Blot Turbo apparatus (Trans-Blot Turbo Transfer System, Bio-Rad, Hercules, California, USA). Subsequently, a blocking step was performed using 3% (w/v) fat-free milk in TBST (50 mM Tris-HCl; 150 mM NaCl, pH 7.4; 0.1% Tween 20) for 30 min. The membrane was then subjected to an overnight incubation at 4 °C with one of the primary antibodies: recombinant anti-BDNF antibody (EPR1292), recombinant anti-NF-kB p65 antibody (EP2161Y), or anti-beta tubulin antibody, which served as the loading control. The following morning, the primary antibody was taken out, and washing and blocking procedures were undertaken for 15 and 30 min, respectively. Subsequently, the goat anti-rabbit IgG H&L (HRP) secondary antibody was applied for 90 min. Refer to Table 2 for a list of antibodies used in this experiment. After 90 min, the secondary antibody was removed, and the membrane underwent a 15 min washing process in preparation for imaging using a ChemiDoc Imaging System (Bio-Rad). Band analysis was conducted using Image Lab 6.0.1, and the findings are expressed as a percentage of the protein/β-tubulin ratio relative to the control group in each gel run, where the control group was normalized to 100% [39,45,46].

### 4.7. Statistical Analysis

The in vivo data are presented using means and standard error of the mean (SEM). Behavioral assessments were examined through two-way repeated-measure analysis of variance (ANOVA) and subsequent Bonferroni multiple-comparison tests. For comparing relative mRNA and protein expression, one-way ANOVA was applied, and subsequent Tukey multiple-comparison tests were conducted. Relative mRNA and protein expression levels in the control and GT NP groups were evaluated employing an unpaired t-test. All statistical analyses were performed in GraphPad Prism 9.0, with a significance level fixed at *p* < 0.05.

## 5. Results

### 5.1. Preparation of NPs from Green Tea Polyphenols via Oxidative Coupling Assembly

**Figure 2:**
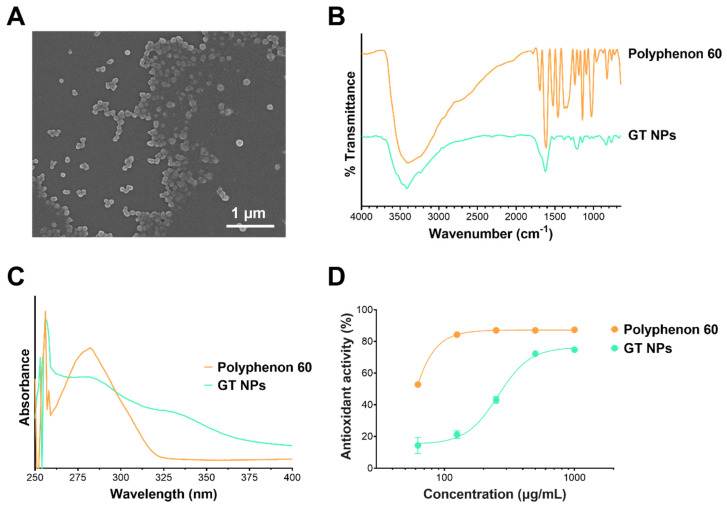
Characterization of GT NPs by (**A**) SEM, (**B**) FT-IR, (**C**) UV-vis, and (**D**) DPPH radical scavenging activity.

Antioxidant NPs from green tea polyphenols were prepared by reacting with CuSO_4_ at 50 °C. The process produced highly monodisperse NPs with a mean hydrodynamic diameter of 191 nm, an average polydispersity index (PDI) of 0.16, and a partially negative surface charge. The NPs exhibited a spherical morphology when examined using SEM (Figure 2A). The FT-IR spectrum of GT NPs was similar to Polyphenon 60 (Figure 2B), albeit with a reduction in broadening of the –O-H stretching band between 3600 and 2700 cm^−1^. The UV spectrum of GT NPs revealed a broadened peak at 282 nm, and increased baseline absorbance attributed to the turbidity of the colloidal NP dispersion (Figure 2C). Antioxidant activity of GT NPs was measured by a DPPH assay and was compared to unreacted Polyphenon 60. As shown in Figure 2D, the process of NP formation by oxidative coupling resulted in a decrease in antioxidant activity of GT NPs compared to unreacted Polyphenon 60. Nonetheless, the NPs maintained significant radical scavenging activity. The concentration required for 50% antioxidant activity (EC_50_) was found to be 39 and 259 µg/mL for Polyphenon 60 and GT NPs, respectively.

### 5.2. Impact of Tobacco Smoke Exposure and GT NP Intervention on Anxiety-like Behavior

**Figure 3:**
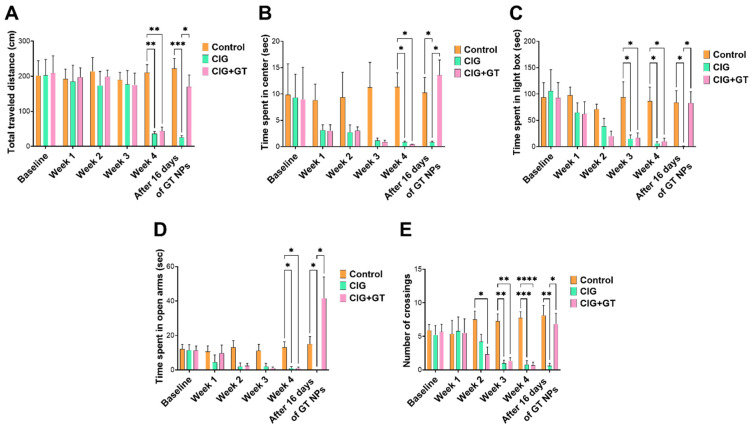
Results of the behavioral tests carried out on the animals before (baseline) and throughout tobacco smoke exposure. (**A**) Distance travelled in the open field (OF); (**B**) time spent in the center of the OF; (**C**) time spent in the illuminated compartment of the light and dark box (LDB); (**D**) time spent in the open arms of the elevated plus maze (EPM); (**E**) number of crossings of the EPM. Data presented as means ± SEM (*n* = 6); * *p* < 0.05, ** *p* < 0.01, *** *p* < 0.001, and **** *p* < 0.0001 based on two-way ANOVA followed by Bonferroni’s multiple-comparison test. Results show increased anxiety following 4 weeks of cigarette smoke exposure, and 16 days’ treatment with GT NPs attenuated this effect.

As outlined in Figure 1, The CIG and CIG+GT animal groups underwent a continuous 4-week exposure to cigarette smoke following the recording of baseline behavior. Figure 3 illustrates that anxiety-like behavior began to manifest in the third or fourth week of exposure. In order to maintain consistent anxiety-like behavior throughout all behavioral tests, the exposure duration was prolonged to 4 weeks. Figure 3A,B present the outcomes of the OF test (the total distance travelled and the duration spent in the center zone). In comparison to the control group, both the CIG and CIG+GT groups exhibited a decline in total distance travelled and time spent in the center zone over the 4-week exposure period. The decline observed in the CIG+GT group was reversed over the 16-day period upon the administration of GT NPs. Regarding the total distance travelled, two-way repeated measure ANOVA revealed significant effects of day (F(5, 80) = 5.818, *p* = 0.0001) and intervention (F(2, 16) = 3.811, *p* = 0.0443), as well as a significant day × intervention interaction (F(10, 80) = 3.941, *p* = 0.0002). Post hoc analysis using Tukey’s multiple-comparison test highlighted a significant decrease in the total travelled distance at week 4 in the CIG and CIG+GT groups in comparison with the control group. This reduction was counteracted by administering 16 daily doses of GT NPs, leading to a significant elevation in the distance travelled in the CIG+GT group in comparison with the CIG group (Figure 3A). This suggests the restoration of locomotion with the administration of GT NPs. As for the time spent in the center of the OF, repeated-measure two-way ANOVA showed no significant main effect of day (F(5, 80) = 3.928, *p* = 0.0355), no significant effect of intervention (F(2, 16) = 3.178, *p* = 0.0386), and no significant day × intervention interaction (F(10, 80) = 2.518, *p* = 0.0093). Post hoc analysis using Tukey’s multiple-comparison test highlighted a significant decrease in the time spent in the center at week 4 in the CIG and CIG+GT groups in comparison with the control group. This reduction was counteracted by administering 16 daily doses of GT NPs, leading to a significant elevation in the time spent in the center in the CIG+GT group in comparison with the CIG group (Figure 3B). Animals subjected to the 16-day treatment with 20 mg/kg GT NPs without prior exposure to cigarette smoke exhibited no significant variation in the total travelled distance or time spent in the center zone of the open field in comparison with the control group (Figure S1, ESI).

In the LDB test, the gradual emergence of withdrawal-induced anxiety was observed over the initial 4 weeks following exposure to tobacco smoke, as indicated by the duration spent in the illuminated compartment (Figure 3C). This pattern mirrored the findings observed in the OF test and was reversed by GT NPs. A significant main effect of day (F(5, 80) = 6.357, *p* < 0.0001), a non-significant main effect of intervention (F(2, 16) = 2.937, *p* = ns), and a significant day × intervention interaction (F(10, 80) = 2.447, *p* = 0.0133) were revealed through repeated-measure two-way ANOVA. Bonferroni’s multiple-comparison test indicated a significant decline in the time spent in the illuminated compartment during weeks 3 and 4 in the CIG and CIG+GT groups in comparison to the control group. This effect diminished after 16 daily doses of 20 mg/kg GT NPs. Additionally, a significant variation in the time spent in the illuminated compartment of the LDB was observed in the CIG group in comparison with the CIG+GT and control groups (Figure 3C). Aligning with the OF test, administration of 20 mg/kg of GT NPs for 16 days with no tobacco smoke exposure revealed no significant variation in the time spent in the illuminated compartment in the LDB in comparison with the control group (Figure S1, ESI).

Likewise, in the EPM test, anxiety induced by tobacco smoke withdrawal manifested over the initial 4 weeks, as evidenced by the duration spent in the open arms and the number of crossings (Figure 3D and Figure 3E, respectively). GT NPs effectively alleviated this anxiety-inducing effect. Concerning the duration spent in open arms, repeated-measure two-way ANOVA demonstrated a significant main effect of day (F(5, 80) = 6.428, *p* = 0.0048), a non-significant effect of intervention (F(2, 16) = 3.339, *p* = ns), and a significant day × intervention interaction (F(10, 80) = 6.102, *p* < 0.0001). Bonferroni’s multiple-comparison test revealed a significant decline in the duration spent in open arms during week 4 in the CIG and CIG+GT groups in comparison with the control group. After 16 days, the CIG group displayed a significant variation in the time spent in open arms in comparison with the CIG+GT and control groups (Figure 3D). A comparable result was noted regarding the number of crossings. Repeated-measure two-way ANOVA showed a significant main effect of day (F(5, 80) = 2.763, *p* < 0.048), intervention (F(2, 16) = 8.172, *p* = 0.0036), and day × intervention interaction (F(10, 80) = 3.571, *p* = 0.0006). Bonferroni’s multiple-comparison test established a significant variation between the CIG and CIG+GT groups in comparison with the control group during weeks 3 and 4. This effect was alleviated after 16 days of GT NP administration. Simultaneously, a significant variation in the number of crossings was observed in the CIG group in comparison with the CIG+GT and control groups (Figure 3E). Moreover, intervention with GT NPs for 16 days without exposure to tobacco smoke demonstrated no significant variation in the time spent in open arms and the number of crossings in the EPM in comparison with the control group (Figure S1, ESI).

### 5.3. Impact of Tobacco Smoke Exposure and GT NP Intervention on nf-κb and bdnf Relative mRNA Expression in the HIP and HYP Brain Subregions

**Figure 4:**
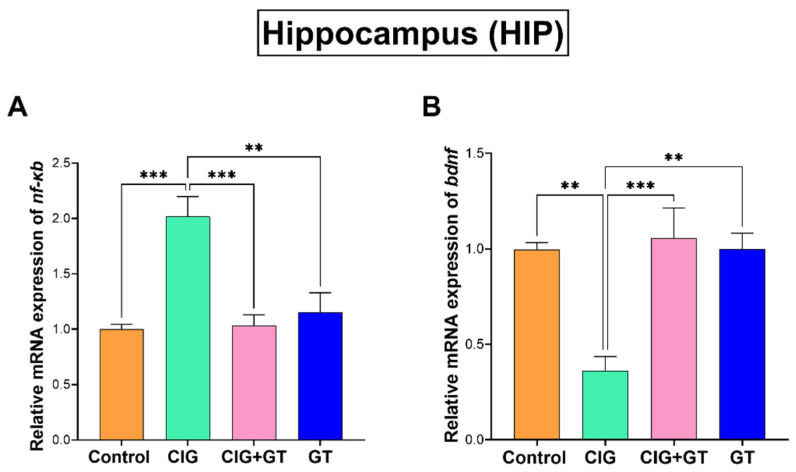
Relative mRNA expression of (**A**) *nf-κb* and (**B**) *bdnf* in the hippocampus (HIP) brain region after tobacco smoke exposure and intervention with GT NPs. Data expressed as means ± SEM (n = 5); ** *p* < 0.01 and *** *p* < 0.001 based on one-way ANOVA followed by Tukey’s multiple-comparison test. Results show a significant increase in the gene expression of *nf-κb* and a significant decrease in *bdnf* gene expression following cigarette smoke exposure, which was attenuated by 16 days of treatment with GT NPs.

Figure 4 illustrates that after 36 days of systemic exposure to tobacco smoke, there was a significant elevation in the relative mRNA expression of *nf-kb* and a significant reduction in the mRNA expression of *bdnf* within the CIG group in comparison with the control, CIG+GT, and GT groups in the HIP. The observed pattern was confirmed by one-way ANOVA, which indicated a significant main effect of intervention on relative *nf-κb* mRNA expression (F(3, 16) = 12.52, *p* = 0.0002, Figure 4A) and a significant main effect of intervention on relative *bdnf* mRNA expression (F(3, 16) = 11.31, *p* = 0.0003; Figure 4B) in the HIP.

**Figure 5:**
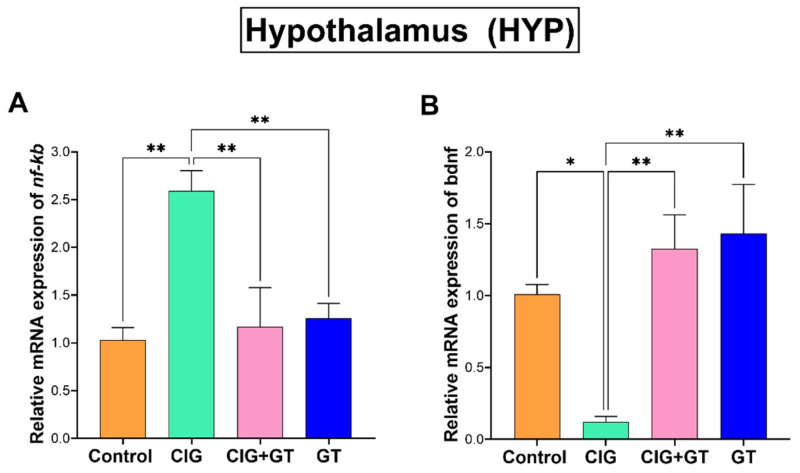
Expression levels of relative mRNA of (**A**) *nf-κb* and (**B**) *bdnf* in the hypothalamus (HYP) brain region after tobacco smoke exposure and intervention with GT NPs. Data expressed as means ± SEM (n = 5); * *p* < 0.05 and ** *p* < 0.01 based on one-way ANOVA followed by Tukey’s multiple-comparison test. Results show a significant increase in the gene expression of *nf-κb* and a significant decrease in *bdnf* gene expression following cigarette smoke exposure, which was attenuated by 16 days of treatment with GT NPs.

As depicted in Figure 5, a 36-day exposure to systemic tobacco smoke led to a significant elevation in the relative *nf-κb* mRNA expression and a significant reduction in relative *bdnf* mRNA expression in the HYP within the CIG group in comparison with the control, CIG+GT, and GT groups. This observed trend is supported by the results of one-way ANOVA, indicating a significant main effect of intervention on relative *nf-κb* mRNA expression (F(3, 16) = 8.341, *p* = 0.0014; Figure 5A) and a significant main effect of intervention on relative *bdnf* mRNA expression (F(3, 16) = 7.876, *p* = 0.0019; Figure 5B) in the HYP.

### 5.4. Impact of Tobacco Smoke Exposure and GT Intervention on Protein Expression Levels of NF-κB and BDNF in the HIP and HYP Brain Subregions

**Figure 6:**
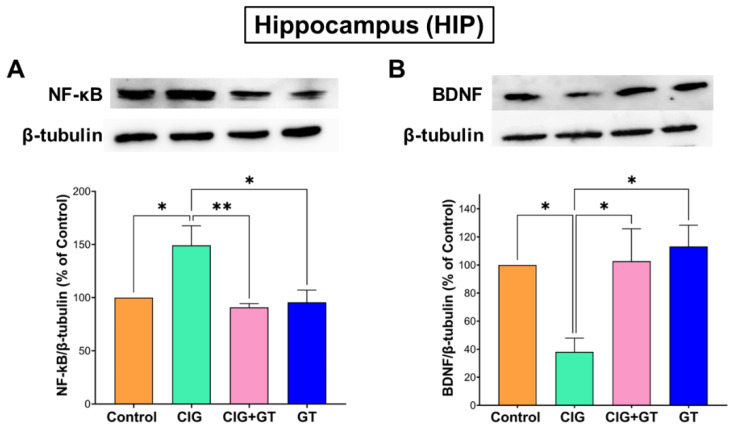
Protein expression of (**A**) NF-κB and (**B**) BDNF in the hippocampus (HIP) brain region after tobacco smoke exposure and intervention with GT NPs. Data expressed as means ± SEM (n = 5); * *p* < 0.05 and ** *p* < 0.01 based on one-way ANOVA followed by Tukey’s multiple-comparison test. Results show a significant increase in the protein expression of NF-κB and a significant decrease in BDNF protein expression following cigarette smoke exposure, which was attenuated by 16 days of treatment with GT NPs.

The gene expression findings were supported by assessing the protein expression levels of NF-κB and BDNF in both the HIP and HYP. Consistent with the relative mRNA expression results, a 36-day systemic exposure to tobacco smoke resulted in a significant increase in NF-κB levels and a significant decrease in BDNF levels in the HIP within the CIG group in comparison to the control, CIG + GT, and GT groups. This observed outcome was verified by one-way ANOVA indicating a significant intervention effect on NF-κB levels (F(3, 16) = 6.07, *p* = 0.0058; Figure 6A) and BDNF levels (F(3, 16) = 5.416, *p* = 0.0092; Figure 6B) in the HIP.

**Figure 7:**
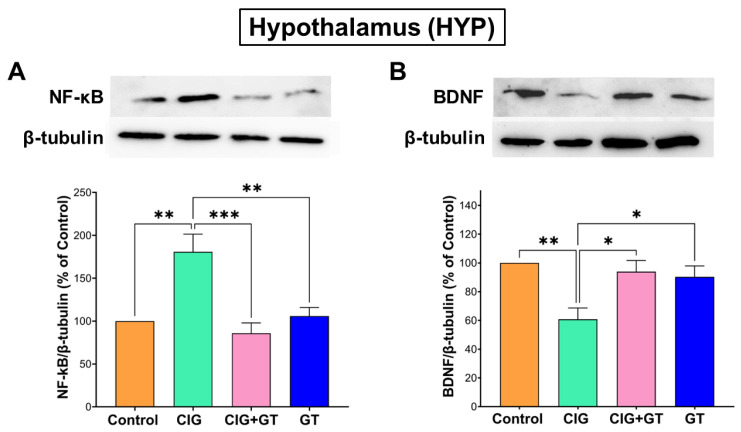
Protein expression of (**A**) NF-κB and (**B**) BDNF in the hypothalamus (HYP) brain region after cigarette smoke exposure and treatment with GT NPs. Data expressed as means ± SEM (n = 5); * *p* < 0.05, ** *p* < 0.01, and *** *p* < 0.001 based on one-way ANOVA followed by Tukey’s multiple-comparison test. Results show a significant increase in the protein expression of NF-κB and a significant decrease in BDNF protein expression following cigarette smoke exposure, which was attenuated by 16 days of treatment with GT NPs.

The protein expression analysis further verified the findings from the analysis of gene expression of *nf-kb* and *bdnf* in the HYP. As shown in Figure 7, a 36-day systemic exposure to tobacco smoke led to a significant elevation in NF-κB levels and a significant reduction in BDNF levels in the HYP within the CIG group, in comparison with the control, CIG+GT, and GT groups. This observed trend was verified by one-way ANOVA, indicating a significant main effect of intervention on NF-κB levels (F(3, 16) = 10.8, *p* = 0.0004; Figure 7A) and on BDNF levels (F(3, 16) = 6.739, *p* = 0.0038; Figure 7B) in the HYP.

## 6. Discussion

A multitude of biological activities linked to green tea polyphenols is complemented by their intriguing chemistry, which has inspired their utilization as the primary source for functional NPs. The intrinsic antioxidant properties of GT NPs have endowed them with similar bioactivities as their small-molecule precursors, with the added advantage of serving as carriers for other therapeutic molecules for anticancer [49,50] and anti-inflammatory applications [51,52]. This study marks the primary exploration into the impact of tobacco smoke exposure and the application of antioxidant-rich GT NPs on anxiety-like behavior and neuroinflammation triggered by smoking withdrawal. The 2 h daily exposure to tobacco smoke over a 36-day span, conducted 5 days a week, led to neuroinflammation. This was evidenced by the amplified relative mRNA and protein expression levels of NF-κB, accompanied by a decline in the relative mRNA and protein expression levels of BDNF in both the HIP and HYP brain subregions.

Exposure to tobacco smoke prompted acute withdrawal-induced anxiety-like behavior, as evidenced by the decreased distance travelled in the OF, reduced duration of time spent in the illuminated compartment in LDB, and diminished duration of time spent in the open arms and the number of crossings in EPM. Notably, the administration of GT NPs effectively reversed these behavioral changes. Furthermore, the NPs alleviated the impact of tobacco smoke exposure on NF-κB and BDNF levels in the mesocorticolimbic brain regions.

In alignment with the findings of this study, previous research conducted by our team and other researchers has consistently reported anxiogenic effects linked to withdrawal from tobacco smoking or nicotine [35,39,46,53,54]. Anxiety is a notable reported behavioral symptom during smoking cessation, surfacing hours or days after individuals quit smoking. This often compels smokers to seek relief by lighting another cigarette. Benzo[a]pyrene diol epoxide, a prominent constituent of cigarette smoke, has been identified as a stimulant for the production of cyclooxygenase 2 (COX-2). This effect occurs, at least to some extent, via its impact on NF-κB in rat astrocytes, observed in both in vitro and in vivo settings [55]. The effects on microglia could serve as a partial mediator for these outcomes. The close connection between oxidative stress and inflammation is widely acknowledged, with oxidative stress assumed to contribute to the regulation of proinflammatory signaling pathways [56]. To emphasize this point, previous studies showed that, like conventional cigarettes, the use of heat-not-burn devices impacts specific signaling pathways that influence the neuroinflammatory processes in different brain regions, including the nucleus accumbens [57,58].

Extended exposure to cigarettes led to oxidative damage, activation of NF-κB, and the generation of proinflammatory compounds [59]. Furthermore, multiple studies have suggested that the synthesis of inflammatory cytokines and the activation of NF-κB play pivotal roles in the cognitive impairment associated with potential cognitive decline [60,61]. Previous studies reported that mice exposed to cigarette smoke had elevated levels of TNF-α, NF-κB p65, and IL-6 [62,63]. Both in vitro and in vivo, exposure to tobacco smoke causes microglial activation and neuronal injury [64]. In line with our results, proinflammatory cytokine levels, such as NF-κB, have been linked to behavioral effects of nicotine withdrawal, such as anxiety [56,61].

Ample evidence describe the advantages of BDNF in boosting neuronal activities (for review, see [65]). BDNF is known to enhance neurogenesis, neural stem cell survival, neuronal differentiation, and other neurophysiological processes. Additionally, BDNF deficiency has been documented to be linked with pathophysiological characteristics of numerous neuropsychiatric illnesses [66,67,68]. Among the various psychiatric and neurological illnesses that BDNF affects is the pathophysiology of anxiety disorders, where it plays a significant role [69]. According to some theories, acute inflammation upregulates BDNF as a defense mechanism for neurons [42]. However, persistent inflammation may result in BDNF downregulation, which may entail maladaptive processes [70]. Additionally, the effect may be specific to certain brain regions. As demonstrated by our study and others, exposure to tobacco smoke elevated BDNF levels in the nucleus accumbens and prefrontal cortex regions of the brain, while simultaneously decreasing BDNF levels in the HIP [46,71]. Moreover, short-term exposure to tobacco smoke decreased serum BDNF levels, but long-term exposure increased serum BDNF levels [72]. Therefore, it would seem that exposure to tobacco smoke causes changes in BDNF that are dependent on brain region, tissue, and duration of exposure. Our results showed that tobacco smoke exposure for 36 days caused an upregulation in NF-κB expression and a downregulation in BDNF expression in the HIP and the HYP of rat brains, and these changes might be the cause of smoking withdrawal anxiety-like behavior induction.

Previous studies demonstrated the anxiolytic effect of green tea and major components in rodent models (for review, see [73]). This is in line with our results, in which intervention with GT NPs for 16 days ameliorated anxiety-like behavior as measured through OF, LDB, and EPM. The administration of EGCG, whether intracerebroventricular or oral, resulted in behavioral effects indicative of anxiolytic activity in a mouse animal model [74,75]. Importantly, EGCG attenuated anxiety-like behavior through an anti-inflammatory effect [76]. A prior study reported that EGCG inhibited the expression of lipopolysaccharide (LPS)-induced inflammatory cytokines in brain endothelial cells [77]. The generation of inflammatory cytokines by LPS is recognized as depending on NF-κB activation [78]. An additional study demonstrated that green tea polyphenols are recognized for their ability to prevent oxidized LDL-induced NF-κB activation [79]. Moreover, in the intestinal epithelial cell line IEC-6, it was found that EGCG blocked NF-κB activation [80]. Conversely, EGCG improved SPS-stimulated memory and behavioral dysregulation in rats, in part through preventing the decrease in BDNF levels [60]. Furthermore, theanine administration, another constituent of green tea extracts, has been revealed to elevate BDNF levels [81,82]. Tea polyphenols prevented staurosporine-induced cytotoxicity, apoptosis and the morphological changes by upregulating the BDNF signaling axis within rat hippocampal neurons [83]. Taken together, these results validate the beneficial effect of green tea polyphenols (especially EGCG) as an anti-inflammatory as well as neuroprotective intervention that boosts the neuritogenic activity of BDNF. These reports align with the current study, in which GT NPs alleviated the harmful effect of tobacco smoke exposure by downregulating NF-κB and upregulating BDNF in both the HIP and HYP.

## 7. Conclusion

This study found that tobacco smoke exposure for 36 days induced anxiety-like behavior as measured through OF, LDB, and EPM, as well as induced neuroinflammation through upregulation of NF-κB and downregulation of BDNF in HIP and HYP brain regions. GT NPs administered for 16 days ameliorated the behavioral dysfunction and restored the levels of NF-κB and BDNF in the HIP and HYP. Our findings highlight the promising role of dietary antioxidants such as tea polyphenols as anxiolytic, anti-inflammatory, and neuroprotective agents that could help smokers quit smoking more effectively. Moreover, GT NPs could serve as nanocarriers for additional therapeutic molecules to achieve synergistic effects in neuroinflammation and other inflammatory contexts.

## Figures and Tables

**Table 1:** Primer sequences analyzed in this study.

Gene	Primer	Sequence	Reference
*bdnf*	Forward	5′-GGCAGGTTCGAGAGGTCTGA-3′	[42]
	Reverse	5′-CGCTGTGACCCACTCGCTAA-3′	
*nf-kb*	Forward	5′-TGGCAGACGACGATCCTTTC-3′	[43]
	Reverse	5′-GAAGGTATGGGCCATCTGTTGA-3′	
*b-actin*	Forward	5′-ATCTGGCACCACACCTTC-3′	[44]
	Reverse	5′-AGCCAGGTCCAGACGCA-3′	

**Table 2:** Antibodies used in this study.

Antibody	Company (cat#)	Host Species	Dilution
Recombinant anti-BDNF antibody [EPR1292] [47]	Abcam, (ab108319)	Rabbit	1/1000
Recombinant anti-NF-kB p65 antibody [EP2161Y]	Abcam, (ab76311)	Rabbit	1/1000
Anti-beta tubulin antibody	Abcam, (ab6046)	Rabbit	1/2000
Goat anti-rabbit IgG H&L (HRP) [48]	Abcam, (ab6721)	Goat	1/1000

All antibodies were purchased from Abcam, Cambridge, UK.

## Data Availability

The original data presented in the study are included in the article/Supplementary Materials; further inquiries can be directed to the corresponding author.

## Supplementary Materials

The following supporting information can be downloaded at https://www.mdpi.com/article/10.3390/toxics12080598/s1. Figure S1: Results of the behavioral tests carried out on animals exposed to room air before (baseline) and after 16 days of GT NPs administration at 20 mg/kg p.o.; Figure S2: Image of the western blot gel resulting from the analysis of NF-κB expression in the hippocampus; Figure S3: Image of the western blot gel resulting from the analysis of BDNF expression in the hippocampus; Figure S4: Image of the western blot gel resulting from the analysis of NF-κB expression in the hypothalamus; Figure S5: Image of the western blot gel resulting from the analysis of BDNF expression in the hypothalamus.
