# Supplementary material for: Green Tea Polyphenol Nanoparticles Reduce Anxiety Caused by Tobacco Smoking Withdrawal in Rats by Suppressing Neuroinflammation
Source: Toxics. 2024 Aug 18;12(8):598. doi: 10.3390/toxics12080598 (PMC11360476; doi:10.3390/toxics12080598)
Supplement: Supplementary file 1 [file toxics-12-00598-s001.zip › toxics-3140977-supplementary.pdf]

Electronic Supplementary Information (ESI)

Green Tea Polyphenol Nanoparticles Reduce Anxiety Caused by Tobacco Smoking Withdrawal in Rats by Suppressing Neuroinflammation †

Alaa M. Hammad [1,*], Lujain F. Alzaghari [1], Malek Alfaraj [1], Vanessa Lux [2] and Suhair Sunoqrot [1,*]

[1] Department of Pharmacy, Faculty of Pharmacy, Al-Zaytoonah University of Jordan, Amman 11733, Jordan

[2] Department of Genetic Psychology, Ruhr University Bochum, 44801 Bochum, Germany; (V.L.) vanessa.lux@rub.de

* Correspondence: alaa.hammad@zuj.edu.jo (A.M.H.); suhair.sunoqrot@zuj.edu.jo (S.S.)

† Electronic Supplementary Information (ESI) available: Results of the behavioral tests of control groups and uncropped western blot gel images.

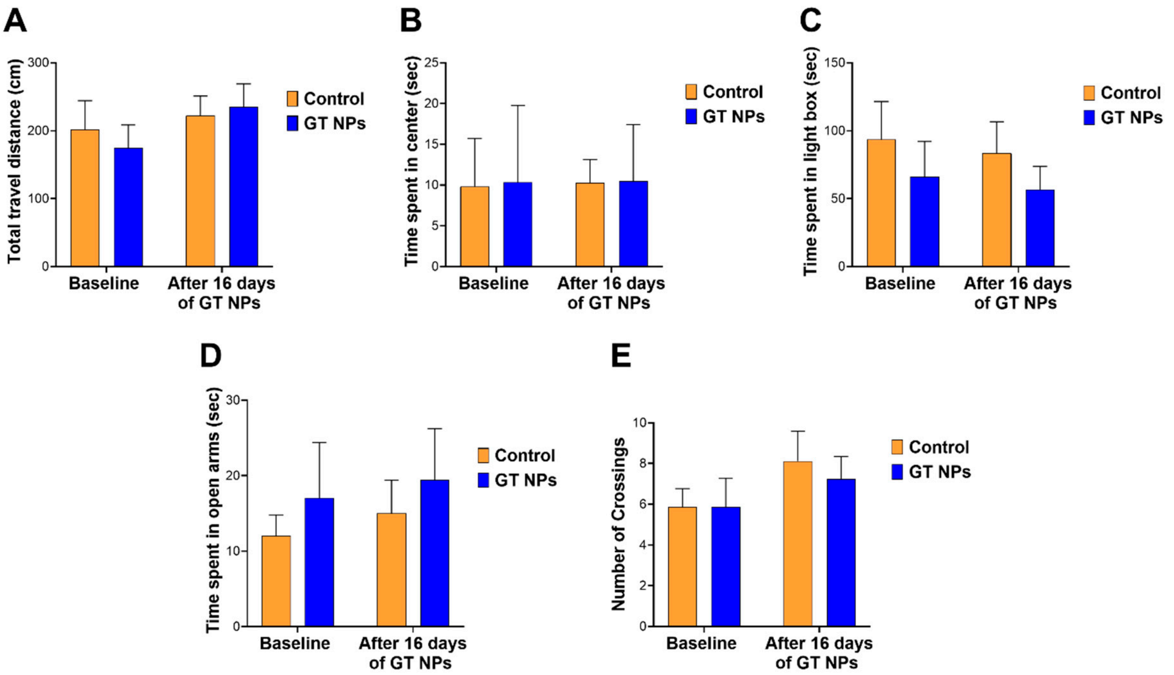

Figure S1. Results of the behavioral tests carried out on animals exposed to room air before (baseline) and after 16 days of GT NPs administration at 20 mg/kg p.o.. (A) Distance travelled in the OF; (B) Time spent in the center of the OF; (C) Time spent in the illuminated compartment of the LDB; (D) Time spent in the open arms of the EPM; (E) Number of crossings of the EPM. Data is presented as mean ± SEM (n=6). No difference was found between the control and GT NPs groups based on one-way ANOVA followed by Tukey's multiple comparisons test.

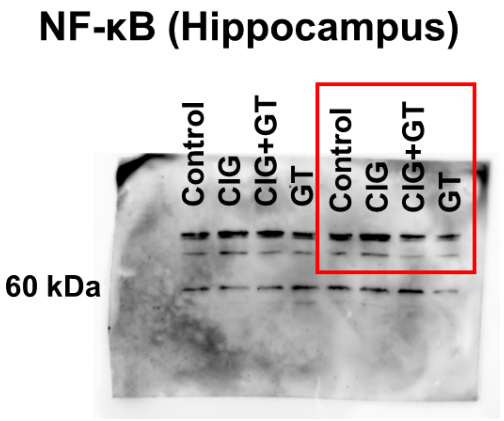

Figure S2. Image of the western blot gel resulting from the analysis of NF-κB expression in the hippocampus. Lanes surrounded by the red border correspond to the image presented in Figure 6A of the main manuscript. Control: group exposed to a standard air environment; CIG: cigarette smoke-exposed group; CIG+GT: cigarette smoke-exposed group which received green tea nanoparticles (GT NPs) treatment; GT: group exposed to a standard air environment which received GT NPs treatment.

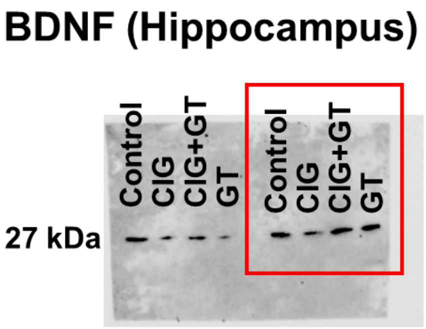

Figure S3. Image of the western blot gel resulting from the analysis of BDNF expression in the hippocampus. Lanes surrounded by the red border correspond to the image presented in Figure 6B of the main manuscript. Control: group exposed to a standard air environment; CIG: cigarette smoke-exposed group; CIG+GT: cigarette smoke-exposed group which received green tea nanoparticles (GT NPs) treatment; GT: group exposed to a standard air environment which received GT NPs treatment.

NF-κB (Hypothalamus)

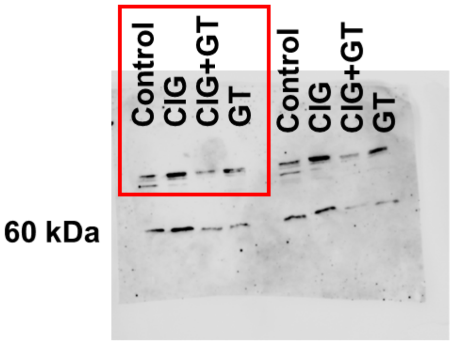

Figure S4. Image of the western blot gel resulting from the analysis of NF-κB expression in the hypothalamus. Lanes surrounded by the red border correspond to the image presented in Figure 7A of the main manuscript. Control: group exposed to a standard air environment; CIG: cigarette smoke-exposed group; CIG+GT: cigarette smoke-exposed group which received green tea nanoparticles (GT NPs) treatment; GT: group exposed to a standard air environment which received GT NPs treatment.

BDNF (Hypothalamus)

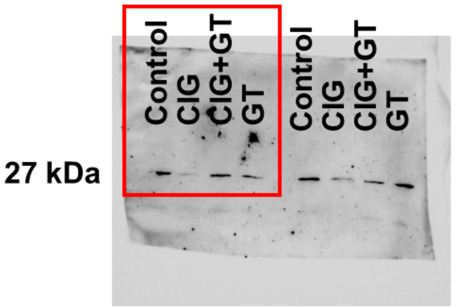

Figure S5. Image of the western blot gel resulting from the analysis of BDNF expression in the hypothalamus. Lanes surrounded by the red border correspond to the image presented in Figure 7B of the main manuscript. Control: group exposed to a standard air environment; CIG: cigarette smoke-exposed group; CIG+GT: cigarette smoke-exposed group which received green tea nanoparticles (GT NPs) treatment; GT: group exposed to a standard air environment which received GT NPs treatment.
